# The characteristics and risk factors of in-stent restenosis in patients with percutaneous coronary intervention: what can we do

**DOI:** 10.1186/s12872-020-01798-2

**Published:** 2020-12-04

**Authors:** Pengfei Wang, Haixia Qiao, RuiJuan Wang, Ruitian Hou, Jingtao Guo

**Affiliations:** 1 Department of Cardiology, Affiliated Hospital of Chengde Medical College, No. 36 Nanyingzi Street, Chengde, 067000 Hebei Province People’s Republic of China; 2 Department of Cardiology, Chengde Central Hospital, Chengde, People’s Republic of China

**Keywords:** In-stent restenosis, PCI, Cardiovascular, Risk, Prevention, Nursing

## Abstract

**Background:**

Percutaneous coronary intervention (PCI) is a common treatment for patients with coronary heart disease, and intra-stent restenosis (ISR) is a serious complication after PCI. It’s necessary to identify the potential risk factors to provide evidence for the prevention of ISR.

**Methods:**

The patients who underwent coronary angiography 1 year after PCI in our hospital from January 2017 to May 2019 were selected. The characteristics and results of clinical examination of ISR and no-ISR patients were compared, Multivariate logistic regression analyses were performed to identify the risk factors.

**Results:**

A total of 209 patients were included, the incidence of ISR after PCI was 30.62%. There were significant differences on the hypertension, diabetes, number of coronary artery lesions, reasons for stent implantation, the diameter of stent, the length of stent and stent position between ISR group and no-ISR patients (all *p* < 0.05). The LDL-C in ISR groups was significantly higher than that of no-ISR group (*p* = 0.048), there were no significant differences between two groups in FPG, TG, TC, HDL-C, Apo A1, Apo B, LP-a and glycated haemoglobin (all *p* > 0.05). The hypertension (OR 4.30, 95% CI 1.12–9.34), diabetes (OR 5.29, 95% CI 1.25–9.01), number of coronary artery lesions ≥ 2 (OR 4.84, 95% CI 1.21–9.55), LDL-C ≥ 1.9 mmol/L (OR 5.93, 95% CI 2.29–10.01), unstable angina (OR 2.92, 95% CI 1.20–4.55), left anterior descending artery (OR 4.01, 95% CI 1.73–7.58), diameter of stent ≥ 3 mm (OR 5.42, 95% CI 1.24–10.84), the length of stent > 20 mm (OR 3.06, 95% CI 1.19–5.22) were the independent risk factor for ISR (all *p* < 0.05).

**Conclusion:**

It is necessary to take preventive measures against these risk factors to reduce ISR, and studies with larger sample size and longer follow-up on this issue are needed in the future.

## Summary graph of the results

- The incidence of ISR was 30.62%.
- The LDL-C in ISR groups was significantly higher than that of no-ISR group.
- The hypertension, diabetes, number of coronary artery lesions ≥ 2, LDL-C ≥ 1.9 mmol/L, unstable angina, left anterior descending artery, diameter of stent ≥ 3 mm, the length of stent > 20 mm were independent risk factor for ISR.

## Background

With the continuous improvement of life quality, the global cardiovascular disease prevalence is in a continuous rising stage, and the mortality of cardiovascular disease ranks first, which is higher than that of tumors and other diseases [1, 2]. The proportion of died patients with coronary heart disease (CHD) among the total number of deaths has increased from 8.6% in 1990 to 15.2% in 2013 [3]. With the intensification of the aging process, the morbidity and mortality of CHD in various countries will continue to increase, which seriously threatens the health of the people and brings a heavy social and economic burden [4]. Therefore, the prevention and treatment of coronary heart disease is extremely important.

Percutaneous coronary intervention (PCI) is one of the main treatments for patients with CHD, and intra-stent restenosis (ISR) after PCI has gradually attracted clinical attention [5]. ISR refers to the plaque within 5 mm of the edge of the stent after PCI and has a stenosis greater than 50% [6]. The incidence of ISR for stents is 15–30% [7]. With the development of interventional technology, the comprehensive application of drug-eluting stents and anti-proliferative drugs such as rapamycin, everolimus, etc. has improved the success rate of interventional therapy [8], but the ISR problem has not been completely resolved. Therefore, it is very urgent to identify the ISR related risk factors to provide evidence for the prevention of ISR. We attempted to evaluate the characteristics and risk factors of ISR in patients with PCI, to provide insights into the prophylaxis and treatment of ISR.

## Methods

### Ethical consideration

This study was reviewed and approved by the Medical Ethics Committee of our hospital (No. 2017011021), and all the included patients had signed written informed consent.

### Patients

The patients who underwent coronary angiography 1 year after PCI in our hospital from January 2017 to May 2019 were selected as the potential research subjects. All patients underwent PCI due to coronary artery stenosis > 75%. The included patients were: adult patients with age > 18 years; it’s the first time for stent implantation; the patients were well-informed and agree to participant in this study. The exclusion criteria were: Patients who were not implanted for the first time or had coronary artery bypass grafting; patients with malignant tumors, autoimmune diseases, and severe liver and kidney dysfunction; patients with cardiomyopathy, congenital heart disease, and valvular disease; patients who did not willing to participant in this study.

### Interventions

All patients received conventional treatment for coronary heart disease, including reasonable diet, body weight control, smoking and alcohol cessation, moderate exercise, maintaining emotional stability, and rational use of drugs. All received oral aspirin (AstraZeneca Pharmaceutical Co., Ltd., H32026199, 0.1 g), the initial dose was 300 mg, and then 100 mg/time, once a day; at the same time, ticagrelor tablets (AstraZeneca Pharmaceutical Co., Ltd., J20171077, 90 mg), the initial dose was 180 mg, and then 90 mg once for every 12 h.

### ISR diagnosis

All patients underwent coronary angiography 1 year after surgery. Left anterior, right anterior and head-foot axis projections were used to observe coronary artery lesions, and the degree of stenosis of the left main trunk, left anterior descending branch, right coronary artery and left circumflex branch lesions were accurately evaluated. For the coronary artery angiography records, two interventional physicians were arranged to complete the corresponding diagnosis independently. If there were any disagreement in the diagnosis, a third doctor was referred. ISR was defined as the narrowing of the lumen diameter of the target vessel ≥ 50%, including the coronary arteries in the stent and the ≤ 5 mm area of the stent [9].

### Data collection

We developed a uniformed form to collect following information for every included patients: (1) The characteristics of patients, including gender, age, drinking history, smoking history, antiplatelet drug use status (such as aspirin, clopidogrel, etc.), and history of diabetes and hypertension. (2) The results of clinical examination, including fasting blood glucose (FPG), triglyceride (TG), total cholesterol (TC), low density lipoprotein cholesterol (LDL-C), high density lipoprotein cholesterol (HDLC), apolipoprotein A1 (Apo A1), apolipoprotein B (Apo B), lipoprotein a (LP-a), glycated haemoglobin. (3) The PCI details, including the reasons for stent implantation, which was divided into stable angina, unstable angina, and acute myocardial infarction. The number of coronary artery lesion. The details of stent implantation, including implantation position, which was divided into left anterior descending artery, left circumflex artery and right coronary artery, the stent length (≤ 20 mm or > 20 mm), stent diameter (< 3 mm or ≥ 3 mm). Regular taking antiplatelet drugs after surgery was defined as regular oral administration of aspirin or clopidogrel after operation, and any one or both of them shouldn’t be discontinued for more than 3 days.

### Statistical analysis

All of the statistical analyses were conducted using SPSS 23.0 (SPSS Inc., Chicago, USA). Categorical variables were analyzed using the χ^2^ test or Fisher’s exact test, and continuous variables were analyzed using Student’s t test. Multivariate logistic regression analyses were performed using the forward likelihood ratio selection method to identify independent risk factors. All the P values were two-tailed, and *p* < 0.05 was considered as being statistically significant.

## Results

### The characteristics of included patients

A total of 209 patients were included, of which 64 patients were diagnosed as ISR, the incidence of ISR was 30.62%. as Table 1 presented, there were significant differences on the hypertension, diabetes, number of coronary artery lesions, reasons for stent implantation, the diameter of stent, the length of stent and stent position between ISR group and no-ISR group (all *p* < 0.05). But no significant difference on the gender, age, history of smoke and drinking, number of implanted stents, and antiplatelet drugs regularly taking between two groups were found (all *p* > 0.05).

**Table 1:** The characteristics of included patients

Variables	ISR group (n = 64)	No-ISR group (n = 145)	t/χ^2^	*p*
Male/female	42/22	97/48	1.204	0.117
Age (years)	65.39 ± 8.91	64.04 ± 9.12	7.226	0.052
Hypertension	43 (67.19%)	55 (37.93%)	1.127	0.009
Diabetes	36 (56.25%)	59 (40.69%)	1.014	0.037
History of smoke	22 (34.38%)	49 (33.79%)	1.205	0.089
History of drinking	19 (29.69%)	40 (27.59%)	1.184	0.092
Number of coronary artery lesions				
One artery	25 (39.06%)	96 (66.21%)	1.947	0.022
Two arteries	24 (37.50%)	44 (30.34%)		
Three arteries	15 (23.44%)	5 (3.45%)		
Reasons for stent implantation				
Stable angina	12 (18.75%)	25 (17.24%)	1.374	0.016
Unstable angina	36 (56.25%)	44 (30.34%)		
Acute myocardial infarction	16 (25%)	76 (52.42%)		
Number of implanted stents (n)	2.31 ± 1.05	2.01 ± 1.10	1.294	0.117
The diameter of stent				
< 3 mm	38 (59.38%)	61 (42.07%)	1.331	0.028
≥ 3 mm	26 (40.62%)	84 (57.93%)		
The length of stent				
≤ 20 mm	4 (6.25%)	58 (40%)	1.097	0.034
> 20 mm	60 (93.75%)	87 (60%)		
Stent position				
Left anterior descending artery	36 (56.25%)	54 (37.24%)	1.164	0.027
Left circumflex artery	8 (12.50%)	32 (22.07%)		
Right coronary artery	20 (31.25%)	59 (40.69%)		
Take antiplatelet drugs regularly	37 (57.81%)	80 (55.17%)	1.089	0.085

### The results of clinical examination of included patients

As Table 2 presented, the LDL-C in ISR groups was significantly higher than that of no-ISR group (*p* = 0.048), there were no significant differences between two groups in FPG, TG, TC, HDL-C, Apo A1, Apo B, LP-a and glycated haemoglobin level (all *p* > 0.05).

**Table 2:** The results of clinical examination of included patients

Variables	ISR group (n = 64)	No-ISR group (n = 145)	T	*p*
FPG (mmol/L)	7.19 ± 2.05	6.79 ± 2.12	1.275	0.093
TG (mmol/L)	1.54 ± 1.02	1.64 ± 1.07	1.460	0.106
TC (mmol/L)	4.36 ± 0.92	3.41 ± 0.99	1.204	0.053
LDL-C (mmol/L)	2.58 ± 0.41	2.01 ± 0.33	1.129	0.048
HDL-C (mmol/L)	1.03 ± 0.27	1.02 ± 0.21	1.044	0.117
Apo A1 (g/L)	0.95 ± 0.21	0.96 ± 0.18	1.940	0.325
Apo B (g/L)	0.72 ± 0.14	0.70 ± 0.13	1.282	0.113
LP-a (mg/L)	224.73 ± 59.83	232.05 ± 64.11	18.405	0.126
Glycated haemoglobin (%)	5.85 ± 0.74	5.66 ± 0.62	1.204	0.094

### Logistic multi-factor regression analysis

Table 3 indicated the assignment of logistic regression analysis. As Table 4 showed, the hypertension (OR 4.30, 95% CI 1.12–9.34), diabetes (OR 5.29, 95% CI 1.25–9.01), number of coronary artery lesions ≥ 2 (OR 4.84, 95% CI 1.21–9.55), LDL-C ≥ 1.9 mmol/L (OR 5.93, 95% CI 2.29–10.01), unstable angina (OR 2.92, 95% CI 1.20–4.55), left anterior descending artery (OR 4.01, 95% CI 1.73–7.58), diameter of stent ≥ 3 mm (OR 5.42, 95% CI 1.24–10.84), the length of stent > 20 mm (OR 3.06, 95% CI 1.19–5.22) were independent risk factor for ISR (all *p* < 0.05).

**Table 3:** The assignment of logistic multi-factor regression analysis

Variables	Assignment
Hypertension	Yes = 1, no = 0
Diabetes	Yes = 1, no = 0
LDL-C (mmol/L)	≥ 1.9 mmol/L = 1, < 1.9 mmol/L = 0
Number of coronary artery lesions	≥ 2 arteries = 1, 1 artery = 0
Reasons for stent implantation	Unstable angina = 1, other reasons = 0
Stent position	Left anterior descending artery = 1, left circumflex artery = 0
The diameter of stent	≥ 3 mm = 1, < 3 mm = 0
The length of stent	> 20 mm = 1, ≤ 20 mm = 0

**Table 4:** The results of logistic multi-factor regression analysis

Factors	β	S${\overline{\text{x}}}$	OR	95%CI	*p*
Hypertension	0.83	0.22	4.30	1.12–9.34	0.035
Diabetes	0.96	0.33	5.29	1.25–9.01	0.029
Number of coronary artery lesions ≥ 2 arteries	0.88	0.21	4.84	1.21–9.55	0.024
LDL-C ≥ 1.9 mmol/L	0.79	0.25	5.93	2.29–10.01	0.016
Unstable angina	0.59	0.18	2.92	1.20–4.55	0.037
Left anterior descending artery	0.77	0.29	4.01	1.73–7.58	0.044
Diameter of stent ≥ 3 mm	0.96	0.32	5.42	1.24–10.84	0.038
The length of stent > 20 mm	0.79	0.24	3.06	1.19–5.22	0.023

## Discussions

The mechanism of ISR has not been fully elucidated, the results of study have found that the hypertension, diabetes, number of coronary artery lesions ≥ 2 arteries, LDL-C ≥ 1.9 mmol/L, unstable angina, left anterior descending artery, diameter of stent ≥ 3 mm, the length of stent > 20 mm were independent risk factors for ISR in patients with PCI, targeted preventions should be conducted based on those factors. PCI is currently the main effective treatment option for CHD, especially drug-coated stents have greatly improved the efficacy and prognosis of patients with CHD [10]. Although drug-coated stents can significantly reduce the risk of ISR, the risk of acute heart disease events such as progressive angina or acute myocardial infarction caused by ISR still exists, which seriously affects the outcome after PCI [11]. ISR is the formation of endometrial hyperplasia mediated by biological, mechanical, technical and complex factors related to the patient, which will lead to the loss of target blood vessels and increase heart disease events [12]. Therefore, early recognition and prevention of ISR is particularly important.

Diabetes and hypertension patients have relatively complicated vascular lesions and unstable endothelial function [13]. Therefore, during PCI treatment, coronary intimal tear or dissection is more likely to occur, which promotes platelet adhesion [14]. Besides, glucose can also directly damage coronary endothelial cells, these factors increase the incidence of ISR [15]. Unbalanced glucose metabolism activates vascular endothelial inflammation, which is a risk factor for the formation of coronary atherosclerotic plaques [16]. The patient's poor blood glucose control after stenting will aggravate the process of protein glycosylation and oxidation, damaging the vascular endothelium and initiate atherosclerotic plaque, which is related to ISR [17]. Hypertension is a risk factor for CHD. When the blood pressure fluctuates or continues to be under high pressure, the blood accelerates the shear force on the tube wall, damages the vascular endothelial cells [18], and increases the incidence of ISR. Therefore, patients with hypertension must strictly control blood pressure after surgery. For patients with a history of diabetes or hypertension, strictly controlling blood pressure and sugar is beneficial to reduce the risk of ISR.

High LDL-C is an important risk factor for CHD [19]. High levels of LDL-C stimulate inflammation, damage vascular endothelial cells, and promote the deposition of cholesterol in the blood vessel wall [20]. It’s been reported that controlling cholesterol intake can reduce the incidence of CHD [21]. Therefore, standardizing medication after PCI is very important. Intensified statin therapy after PCI to reduce LDL-C levels is beneficial to reduce the neointimal hyperplasia [22]. Previous study [23] has reported that the LDL-C level of patients was significantly correlated with ISR, which is consistent with our findings. Long-term oral lipid-lowering drugs may be required after PCI, and LDL-C should be controlled under 1.9 mmol/L.

Studies [24] have reported that the incidence of ISR of anterior descending branch is significantly higher. And the incidence of ISR in the left anterior descending branch is significantly higher than that of left circumflex branch and the right coronary artery. ISR is more likely to occur in those with thinner vascular [25]. Therefore, for anterior descending branch lesions or complex lesions, it is necessary to strictly understand the PCI surgical indications, and use intravascular ultrasound and other vascular imaging techniques to optimize the surgical plan.

The ISR is closely associated with the damage to the intima, which induces intimal hyperplasia, causing loss of the lumen over time [26]. In the process of PCI treatment, whether it is the injury caused by balloon expansion to the intima or the mechanical stimulation generated by the stent, it may exert an activation effect on inflammatory factors, induce local inflammation, and then stimulate smooth muscle cells [27, 28]. The proliferation reaction also causes a certain deposition of extracellular matrix, resulting in thickening of the intima and ISR [29]. Some doctors [30] have pointed out that both the diameter of the blood vessel and the length of the stent have a certain correlation with the ISR. Arteries with smaller diameters are relatively weak in their ability to adapt to neointimal proliferation into the lumen [31]. The thinner blood vessels are implanted with stents and the blood flow rate is slower, and ISR is more likely to occur after PCI. Multiple stents need to be implanted due to diffuse lesions [32]. The total area of the stent metal material in contact with the blood vessel wall is large, which stimulates vascular endothelial hyperplasia and plaque formation, increasing the risk of ISR [33]. This study has found that stent diameter < 3 mm and stent length > 20 mm are risk factors for ISR, suggesting the importance of optimizing PCI. For small vessel disease or diffuse disease, precisely guide the stent placement and reduce the number of stents is necessary to reduce the incidence of ISR.

Several limitations must be concerned in this study, future studies with larger sample size and longer follow-up are needed. Firstly, since this study is a retrospective design, there might be some bias and heterogeneity between the patient’s treatment. Secondly, the sample size was small, it might be not power enough to detect the potential related risk factors, more studies on the risk factors for the ISR are needed in the future. Finally, we only made a follow-up for one year, and we did not have any died patients, the long-term follow-up with different time intervals are needed in the future.

## Conclusions

In conclusion, for patients with PCI, the hypertension, diabetes, number of coronary artery lesions ≥ 2 arteries, LDL-C ≥ 1.9 mmol/L, unstable angina, left anterior descending artery, diameter of stent ≥ 3 mm, the length of stent > 20 mm are independent risk factors for ISR, interventions targeted on those risk factors are warranted to prevent the ISR.

## Data Availability

All data generated or analyzed during this study are included in this published article, the datasets used and analyzed during the current study are available from the corresponding author on reasonable request.

## Abbreviations

CHD: Coronary heart disease
PCI: Percutaneous coronary intervention
ISR: Intra-stent restenosis
FPG: Fasting blood glucose
TG: Triglyceride
TC: Total cholesterol
LDL-C: Low density lipoprotein cholesterol
HDLC: High density lipoprotein cholesterol
Apo A1: Apolipoprotein A1
Apo B: Apolipoprotein B
LP-a: Lipoprotein a
